# Humanization and directed evolution of the selenium-containing *scFv* phage abzyme

**DOI:** 10.1039/c8ra02798f

**Published:** 2018-05-10

**Authors:** Yan Xu, Pengju Li, Jiaojiao Nie, Qi Zhao, Shanshan Guan, Ziyu Kuai, Yongbo Qiao, Xiaoyu Jiang, Ying Li, Wei Li, Yuhua Shi, Wei Kong, Yaming Shan

**Affiliations:** National Engineering Laboratory for AIDS Vaccine, School of Life Sciences, Jilin University Changchun Jilin China shanym@jlu.edu.cn +86 431 85167751 +86 431 89228979; Faculty of Health Sciences, University of Macau Macau China; Key Laboratory for Molecular Enzymology and Engineering, The Ministry of Education, School of Life Sciences, Jilin University Changchun Jilin China

## Abstract

According to the binding site structure and the catalytic mechanism of the native glutathione peroxidase (GPX), three glutathione derivatives, GSH-S-DNP butyl ester (hapten Be), GSH-S-DNP hexyl ester (hapten He) and GSH-S-DNP hexamethylene ester (hapten Hme) were synthesized. By a four-round panning with a human synthetic *scFv* phage library against three haptens, the enrichment of the *scFv* phage particles with specific binding activity could be determined. Three phage particles were selected binding to each glutathione derivative, respectively. After a two-step chemical mutation to convert the serine residues of the *scFv* phage particles into selenocysteine residues, GPX activity could be observed and determined upto 3000 U μmol^−1^ in the selenium-containing *scFv* phage abzyme which was isolated by affinity capture against the hapten Be. Also the *scFv* phage abzymes elicited by different antigens displayed different catalytic activities. After a directed evolution by DNA shuffling to improve the affinity to the hapten Be, a secondary library with GPX activity was created in which the catalytic activity of the selenium-containing *scFv* phage abzyme could be increased 17%. This study might be helpful for new haptens or antigens design to optimize the abzymes with high binding activities and might also provide a novel scheme for GPX mimic candidates for drug development.

## Abstract 2

A novel two-step sequential screening strategy used for the improvement of GPX mimics and other abzymes.

## Graphical abstract

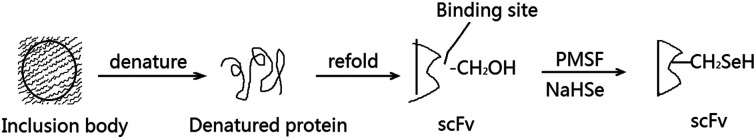

## Introduction

Glutathione peroxidase (GPX) is a key peroxidase for protecting biomolecules from oxidative damage by eliminating hydrogen peroxide and lipid peroxide from the body and further blocking the injury of reactive oxygen free radicals in biological organisms. Selenocysteine is located in the active center of the GPX,^1,2^ which could also reflect the selenium level of the body. Furthermore, selenium, a structural part of a large group of selenoproteins is necessary for proper functioning of the body.^3^ In order to mimic the function of the GPX, numerous studies have been carried out. For example, when hemolyzates from erythrocytes of selenium-deficient rats were incubated *in vitro* in the presence of ascorbate or H_2_O_2_, added glutathione failed to protect the hemoglobin from oxidative damage. This occurred because the erythrocytes were practically devoid of glutathione-peroxidase activity.^4^ Extensively purified preparations of glutathione peroxidase contained a large part of the ^75^Se of erythrocytes labeled *in vivo*. Many of the nutritional effects of selenium can be explained by its role in glutathione peroxidase. Extensive research has been carried out to design and synthesize small organoselenium compounds as functional mimics of GPX. While the catalytic mechanism of the native enzyme itself is poorly understood, the synthetic mimics follow different catalytic pathways depending upon the structures and reactivities of various intermediates formed in the catalytic cycle.^5^ Therefore, GPX plays an important role in antioxidant drugs.

In theory, humanized GPX should be one of the most effective anti-oxidation drugs.^6^ While as the limited source, the poor stability, and the large molecular weight, the application of the native GPX is limited.^7^ Furthermore the native GPX purification is not easy, which could not be conductive to the large-scale preparation.^8^

As a mimic enzyme, the antibody enzyme(abzyme) might possess the catalytic properties, the substrate specificities and the stereo-selectivities as the natural enzymes did. Also, it could be a shortcut that only a few weeks might be taken for an antibody affinity maturation as the incomparable diversity of the abzyme, while millions of years might be taken for a native enzyme evolution. Due to strong binding affinity and high specificity in antibodies against antigens, antibody-catalyzed reactions proceed in a highly regio- and stereo-selective manner, thus the development of new synthetic methods for organic synthesis provides a clear advantage. However, due to the nature of the binding of monoclonal antibody binding to its substrate, the substrate specificity of antibody catalytic reaction is very limited. Therefore, the practical application of catalytic antibodies in organic synthesis requires to expand its substrate specificity.^9^

The abzyme (especially the single-chain abzyme) may have a better stability, lower molecular weight, easier separation and purification process, which could be an ideal medicinal protein. In order to study the catalytic mechanism and develop novel pharmaceutical lead compounds, we have explored several mimic GPXs producing high activity according to the murine abzymes.^10^ Although some abzymes have been reported with efficiencies similar to that of corresponding natural enzymes,^11^ in general it has been difficult to obtain highly active catalysts using the standard approach. As an exogenous protein, the murine GPX enzyme could not be applied in the clinic. It is imperative to develop the humanized GPX abzyme.

The preparation of an abzyme depends on the hapten design. The structure of an ideal hapten candidate should be as close as possible to that of the transition-state analog in the actual reaction, at the same time, as far as possible be different from that of the product and the substrate, which could minimize the inhibition to introduce the active residues involved in the reaction. *In vitro*, there're usually a few approaches utilized to develop humanized hapten-specific antibodies: humanization of a murine antibody, biopanning of a human phage antibody library or a yeast display human *scFv* library, or eliciting antibodies with transgenic animals.

DNA shuffling is a new technique for the directed molecular evolution.^12^ By altering the original nucleotide sequence of a single gene or gene family, a brand new gene could be created and the expression product might display a new function. In recent years, the DNA shuffling technique has been widely applied with broad prospects and great application value in the area of biological engineering.

Phage display is a biotechnology technique that a DNA sequence of foreign proteins or peptides is inserted into the appropriate place of the structural gene of the phage coat protein. The foreign genes could be expressed with the coat protein. At the same time, the foreign proteins could be displayed onto the phage surface with the reassembly of the phage.^13,14^ Phage display technology has been widely applied in the study of protein–protein, protein–peptide, and protein–DNA interactions. Many remarkable advantages have been achieved by phage display technology, such as high flux elutriation, screening of simulant epitope, and easy purification of recombinant phage purification steps.^15,16^ Phage display technology has a great influence on life sciences, and allows users to select extremely rare ligands from the variant library based on user-defined selection criteria.^17^ A large number of studies have been used to find cell-based library selection approaches that many evidences have used to discover chemokine analogs, not only receptor antagonists but also variants with unusual effects on receptor signaling and trafficking.^18^ Thus it becomes a valuable tool for understanding the biology of chemokines.^19^

Phage antibody library technology is also a new genetic engineering antibody technique developed in recent years.^20^ In this technique, the gene fragments of the cloned antibodies *in vitro* were inserted into the phage vector, transfected into the engineering bacteria for the expression, and then the specific monoclonal phage antibody could be obtained by screening against the antigen or haptens. There are unique advantages for application of phage antibody library technology in the diagnosis and treatment of HIV and other viral infections and cancers.^21,22^

In this study, according to the binding site structure and the catalytic mechanism of the native glutathione peroxidase, three glutathione derivatives were synthesized as the haptens representing the maximum level of the basic glutathione structure and the hydrophobicity. After a four-round panning, phage particles recognized specifically by the haptens could be enriched from a human synthetic *scFv* phage library by regulating the concentration of the haptens and controlling the elution strength. It was expected that the binding sites of the antibodies recognized by the haptens could show strong affinities to the glutathione. Furthermore, due to the different modification of the synthesized haptens, the binding site structures of the antibodies might also be different. Then a two-step chemical mutation was employed to convert the serine residues of the *scFv* phage particles into the selenocysteine residues to prepare selenium-containing *scFv* phage abzymes for producing GPX activity.^15^ In order to further improve the activity of the GPX activity abzymes, DNA shuffling was utilized in the sequences of the enriched phage particles with potential GPX activities mimicking the somatic hypermutation occurred *in vivo*.

Comparing the GPX activities of these abzymes, the study might be helpful for new haptens or antigens design to optimize abzymes with high binding activities and might also provide a novel scheme to develop potential GPX mimic candidates for drug development.

## Materials and methods

Human synthetic *scFv* phage library, *E. coli* TG1, Helper phage M13-K07, and Tg1 (pHEN2) were presented by MRC of Cambridge University.

### Synthesis of GSH-S-DNP

1.02 GSH was dissolved in 10 mL 1 mol L^−1^ NaOH solution with ice bath, 0.67 g dinitro chlorobenzene was dissolved in 10 mL ethanol. And then they are added to the GSH solution slowly, the mixture kept in 0 °C 10 minutes, mixed with dilute HCl to pH 4. The yellow crystal precipitation was observed, suction filtered, recrystallized with hot water to 70 °C drying. The final powder is GSH-S-DNP.

### Synthesis of GSH-S-DNP hexyl ester (hapten He) and GSH-S-DNP hexamethylene ester(hapten Hme)

0.21 g GSH-S-DNP was dissolved in 0.2 mL SOCl_2_, mixed with 2 mL *n*-hexylester for 3 hours, then additional 2 mL *n*-hexylester was added and kept at 90 °C for 1 hour. 20 mL ether and 10 mL petroleum ester were added. The yellow precipitation was separated out, frozen for 12 hours, filtered, washed and dried. The final powder is hapten He. The preparation method of hapten Hme is the same as that of hapten He.

### Separation and purification of haptens

As the synthetic derivatives containing the diester and the single ester, we separate them with Gf254 fluorescence silica gel. The liquid phase is chloroform/methanol (v/v = 4 : 1). The first peak at 280 nm was collected and dried. The products are the powder of diesters. The purities were above 95%.

### Enzyme-Linked Immunosorbent Assay (ELISA) and data analysis

The binding activities of the enriched phage particles to the haptens were determined by ELISA. A 96-well plate (Jet Biofil, Guangzhou, China) coated with different haptens was employed for ELISA test. The binding activities of the enriched phage particles to M13-K07 and to the supernatant of TG1 containing pHEN2 were set as the positive control and negative control seperately. The blank is the screening buffer (PBS, 2×TY, TG1, 3% BSA). Optical density measurements were obtained at 450 nm.

The catalytic activities (*U*) of the enriched *scFv* phage particles were calculated as the following equation:
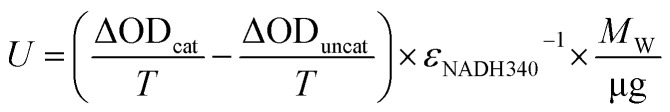


*M*_W_/μg is the micromole of the sample. The micromole of the phage particles is used for calculating the catalytic activity, which is named phage catalytic activity; the unit is U μmol^−1^ phage. The catalytic activity of the pHEN phage particles absent of foreign protein expression is set as the background after converting the serine residues into the selenocysteine residues. The equation for the GPX catalytic activity of the selenium-containing *scFv* phage abzyme is:
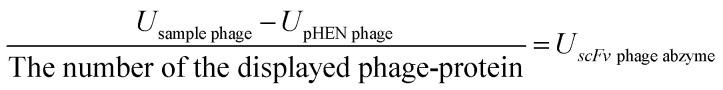


*K*_D_ value was obtained by the relationship between the number of the target protein displayed on the phage particle and the titer of the phage particles; the conversion equation is:*K*_D phage-protein_ = *K*_D phage_/number of the displayed phage-protein

After determined by ELISA, *K*_D_ value of the phage particles was plotted by Graphpad Prism software version 5.0 (Graphpad Software Inc., San Diego, CA, USA).

### DNA shuffling

The DNA mixture of the enriched phage particles with the GPX activity was used as the recombinant PCR template for the directed evolution.

## Results and discussion

### The hapten design

**Fig. 1:**
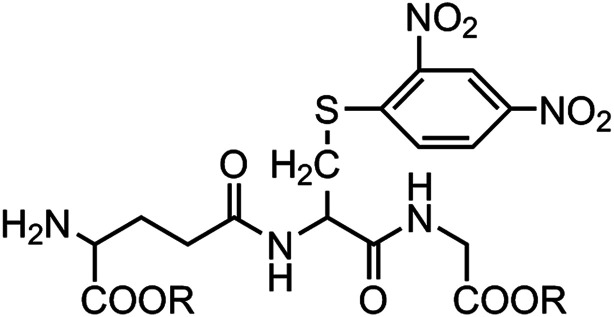
Structures of the haptens used for generating humanized abzymes with potential GPX activity. R: –CH_2_(CH_2_)_2_CH_3_ (hapten Be); –CH_2_(CH)_4_CH_3_ (hapten He); –C_6_H_6_ (hapten Hme).

According to the ability to elicit the murine *scFv* abzyme that is equivalent to the natural enzyme activity,^10^ GSH-S-DNP butyl ester was chosen as the hapten hoping to play the same role in the process of acquiring the humanized GPX mimic. And the hydrophobicities of the three haptens are increasing in order to explore the relationship between the hydrophobicity and the enzyme activity. The three haptens were designed to keep the molecular skeleton characteristics according to the GSH basic structure (Fig. 1). The structures might cause the induced antibodies recognizing the GSH binding sites, but avoid affecting the releasing of the substrate from the enzymatic reaction and further the reaction rate due to the strong specifically binding activity with the GSH. Therefore, it could be possible to obtain a structure of the antibody active site close to the native microenvironment of the GPX catalytic group.

### The enrichment of the specific *scFv* phage particles by affinity screening against haptens with human synthetic *scFv* phage library

Three haptens were screened against the human synthetic *scFv* phage library separately by biopanning.^23^ 50 μg hapten dissolved in 1 mL coating buffer was coated on the Petri dish (Jet Biofil, Guangzhou, China) for the first round screening. And then the following quantity of the hapten was decreased as the increasing round of the screening. The ratio of the hapten amount to the phage particle number was about 1 : 1 for the 1st screening round, and was reduced gradually to 1 : 50 in the following screening rounds. The phage particles with specific binding activity could have been enriched according to the increased recovery ratios as shown in Tables 1–3.

**Table 1:** Affinities of the enriched *scFv* phage against hapten Be

Round	Hapten concentration (μg mL^−1^)	Input phage titer (cfu)	Output phage titer (cfu)	Recovery ratio
1	50	10^13^	1.6 × 10^9^	1.6 × 10^−4^
2	20	10^13^	2.4 × 10^6^	2.4 × 10^−7^
3	10	10^12^	3.1 × 10^6^	3.1 × 10^−6^
4	1	10^12^	1.5 × 10^6^	1.5 × 10^−6^

**Table 2:** Affinities of the enriched *scFv* phage against hapten He

Round	Hapten concentration (μg mL^−1^)	Input phage titer (cfu)	Output phage titer (cfu)	Recovery ratio
1	50	10^13^	7.7 × 10^9^	7.7 × 10^−4^
2	20	10^12^	2.2 × 10^6^	2.2 × 10^−6^
3	10	10^12^	6.8 × 10^6^	6.8 × 10^−6^
4	1	10^12^	2.5 × 10^6^	2.5 × 10^−6^

**Table 3:** Affinities of the enriched *scFv* phage against hapten Hme

Round	Hapten concentration (μg mL^−1^)	Input phage titer (cfu)	Output phage titer (cfu)	Recovery ratio
1	50	10^13^	3.6 × 10^8^	3.6 × 10^−5^
2	20	10^12^	6.5 × 10^6^	6.5 × 10^−6^
3	10	10^12^	6.6 × 10^6^	6.6 × 10^−6^
4	1	10^12^	1.2 × 10^6^	1.2 × 10^−6^

**Fig. 2:**
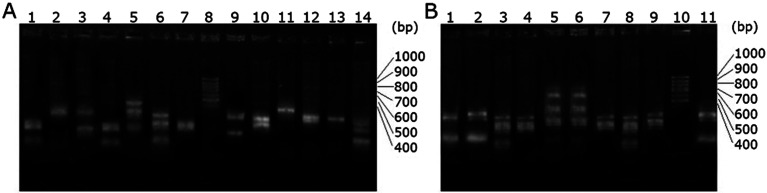
Agarose gel electrophoresis of the *scFv* phage DNA digested with HaeIII. (A) Lane 1–7 and 9–14 are digested native *scFv* phage library DNA, lane 8 is DNA Marker (100 bp Ladder, TRANS, China); (B) Lane 1–9 and 11 are digested enriched *scFv* phage DNA against hapten Be, lane 10 is DNA Marker (100 bp Ladder, TRANS, China).

More than a dozen phage particles were picked and amplified from the enriched *scFv* phage libraries that were specifically binding to the respective haptens. The single strand DNAs were extracted and digested with HaeIII. The electrophoretogram of the digested enriched *scFv*-Be phage DNA is shown in Fig. 2B. The ratio of the screened particles with the similar digested DNA fingerprint patterns has increased obviously compared with that of the original *scFv* phage library (Fig. 2A). This further suggested the enrichment of the specific *scFv* phage particles.

### Catalytic activity determination of the phage particles, Be8, He6 and Hme1 with specific binding activities

The phage particles, which were separately picked from each screened *scFv* phage library after a four-round panning, were recovered by a helper phage M13-K07. The specific binding activities of the recovered phage particles to the haptens were determined by ELISA (Tables 4–6).

**Table 4:** Binding activities of the enriched *scFv* phage particles to the hapten Be

Category	OD_450 nm_	Phage clone number	OD_450 nm_
Positive control	1.621	1, 3, 4, 5, 6, 9, 10	0.1–0.5
Negative control	0.103	7, 11, 12, 13, 15	0.5–1.0
Blank	≤0.085	2, 8, 14	1.0–1.6

**Table 5:** Binding activities of the enriched *scFv* phage particles to the hapten He

Category	OD_450 nm_	Phage clone number	OD_450 nm_
Positive control	1.841	2, 3, 4, 8, 11, 13	0.1–0.5
Negative control	0.160	1, 9, 10, 14, 15	0.5–1.0
Blank	≤0.061	6, 5, 7, 12	1.0–1.6

**Table 6:** Binding activities of the enriched *scFv* phage particles to the hapten Hme

Category	OD_450 nm_	Phage clone number	OD_450 nm_
Positive control	1.506	3, 4, 8, 10, 11, 13, 15	0.1–0.5
Negative control	0.072	6, 2, 5, 7, 9, 12, 14	0.5–1.0
Blank	≤0.004	1	1.0–1.6

Phage particles, Be8, He6 and Hme1, which were the three most positive clones specifically binding to the glutathione derivatives, were purified and sequenced. After determined by ELISA, *K*_D_s of the phage particles were calculated by regression of *Prism*: *K*_D Be8_ = 14 μM; *K*_D He6_ = 12 μM; *K*_D Hme1_ = 12 μM. With the introduction of the selenocysteine residue into the *scFv* phage particles by the two-step chemical mutation, the *scFv* phage abzymes isolated by the affinity capture to the hapten Be could present the catalytic activities from 700 U μmol^−1^ to 3000 U μmol^−1^ similar to that of the native GPX (5780 U μmol^−1^) extracted from rabbit liver.^24–26^

### Directed evolution of the *scFv* sequences

**Fig. 3:**
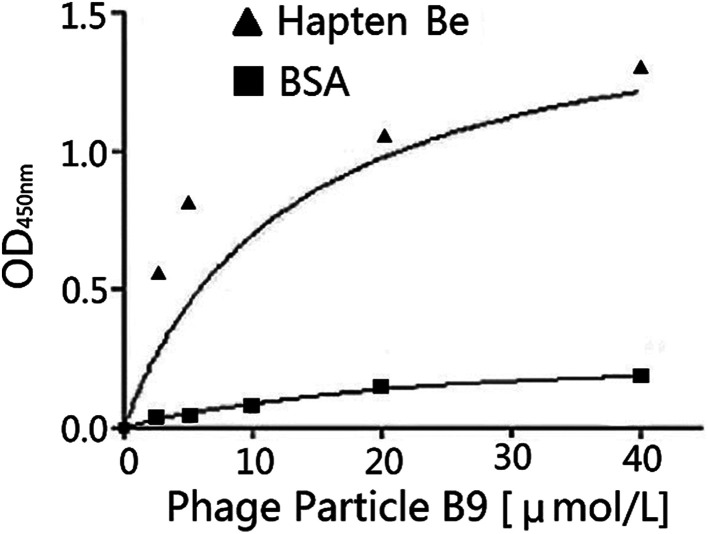
Binding activity of the *scFv* phage particle Be9 after the directed evolution to the immobilized hapten Be7.

A secondary library with GPX activity was created by DNA shuffling. The phage particle affinities to the haptens were improved after the directed evolution^13^ as shown in Table 7. Phage particle B9 was selected for the activity analysis and the *K*_D_ value was calculated after the chemical mutation. The correlative ELISA profile of the phage particle B9 is shown in Fig. 3 and the *K*_D_ value is 13 μM by the regression calculation of *Prism*. The catalytic activity of the selenium-containing *scFv* phage abzyme could reach 3560 U μmol^−1^ according to the measurement of the phage particle activity.^27,28^

**Table 7:** Binding activities of the directed evolution phage particles to the hapten Be

Category	OD_450 nm_	Phage clone number	OD_450 nm_
Positive control	1.537	1, 3, 4, 5, 6, 8, 10, 11	0.1–0.5
Negative control	0.101	2, 7, 12, 13, 14, 15	0.5–1.0
Blank	≤0.061	9	1.0–1.4

## Conclusion

According to the binding site structure and the catalytic mechanism of native glutathione peroxidase, three glutathione derivatives were synthesized in this study. A four-round screening was performed using the MRC human synthetic *scFv* phage library by affinity capture to the respective haptens. The results of the DNA fingerprint analysis and ELISA indicated the enrichment of the phage particles with the specificity to the hapten respectively. After the introduction of the selenocysteine residues into the enriched *scFv* phage particles, the chemical mutated phage abzymes could give the catalytic activities ranging from 700 U μmol^−1^ to 3000 U μmol^−1^. After the directed evolution of these *scFv* phage sequences by DNA shuffling to improve the affinity to the hapten Be, the catalytic activity of the selenium-containing *scFv* phage abzymes could be improved to 3560 U μmol^−1^ similar to that of the native GPX. In short, after bio-panning, a secondary library with GPX activity has been created in which the catalytic activity of the positive phage abzymes could be increased 17% compared with that of either native phage abzymes. The study suggests a novel scheme to develop potential humanized GPX mimic candidates for drug development and might be helpful for new haptens or antigens design to optimize abzymes with high binding and catalytic activities.

## Conflicts of interest

There are no conflicts to declare.

## Supplementary Material
